# Comprehensive target capture/next-generation sequencing as a second-tier diagnostic approach for congenital muscular dystrophy in Taiwan

**DOI:** 10.1371/journal.pone.0170517

**Published:** 2017-02-09

**Authors:** Wen-Chen Liang, Xia Tian, Chung-Yee Yuo, Wan-Zi Chen, Tsu-Min Kan, Yi-Ning Su, Ichizo Nishino, Lee-Jun C. Wong, Yuh-Jyh Jong

**Affiliations:** 1 Department of Pediatrics, Kaohsiung Medical University Hospital, Kaohsiung Medical University, Kaohsiung, Taiwan; 2 Department of Pediatrics, School of Medicine, College of Medicine, Kaohsiung Medical University, Kaohsiung, Taiwan; 3 Baylor Genetics, Houston Texas, United States of America; 4 Department of Molecular and Human Genetics, Baylor College of Medicine, Houston Texas, United States of America; 5 Department of Biomedical Science and Environmental Biology, College of Life Science, Kaohsiung Medical University, Kaohsiung, Taiwan; 6 Department of Pathology, Kaohsiung Medical University Hospital, Kaohsiung Medical University, Kaohsiung, Taiwan; 7 Sofiva Genomics Co., Ltd., Taipei, Taiwan; 8 Dianthus Maternal Fetal Medicine Clinic, Taipei, Taiwan; 9 Department of Obstetrics and Gynecology, School of Medicine, College of Medicine, Taipei Medical University, Taipei, Taiwan; 10 Department of Neuromuscular Research, National Institute of Neuroscience, National Center of Neurology and Psychiatry, Tokyo, Japan; 11 Department of Genome Medicine Development, Medical Genome Center, National Center of Neurology and Psychiatry, Tokyo, Japan; 12 Department of Laboratory Medicine, Kaohsiung Medical University Hospital, Kaohsiung Medical University, Kaohsiung, Taiwan; 13 Graduate Institute of Clinical Medicine, College of Medicine, Kaohsiung Medical University, Kaohsiung, Taiwan; 14 Department of Biological Science and Technology, College of Biological Science and Technology, National Chiao Tung University, Hsinchu, Taiwan; Charite Universitatsmedizin Berlin, GERMANY

## Abstract

**Purpose:**

Congenital muscular dystrophy (CMD) is a heterogeneous disease entity. The detailed clinical manifestation and causative gene for each subgroup of CMD are quite variable. This study aims to analyze the phenotypes and genotypes of Taiwanese patients with CMD as the epidemiology of CMD varies among populations and has been scantly described in Asia.

**Methods:**

A total of 48 patients suspected to have CMD were screened and categorized by histochemistry and immunohistochemistry studies. Different genetic analyses, including next-generation sequencing (NGS), were selected, based on the clinical and pathological findings.

**Results:**

We identified 17 patients with sarcolemma-specific collagen VI deficiency (SSCD), 6 patients with merosin deficiency, two with reduced alpha-dystroglycan staining, and two with striking lymphocyte infiltration in addition to dystrophic change on muscle pathology. Fourteen in 15 patients with SSCD, were shown to have *COL6A*1, *COL6A2* or *COL6A3* mutations by NGS analysis; all showed marked distal hyperlaxity and normal intelligence but the overall severity was less than in previously reported patients from other populations. All six patients with merosin deficiency had mutations in *LAMA2*. They showed relatively uniform phenotype that were compatible with previous studies, except for higher proportion of mental retardation with epilepsy. With reduced alpha-dystroglycan staining, one patient was found to carry mutations in *POMT1* while another patient carried mutations in *TRAPPC11*. *LMNA* mutations were found in the two patients with inflammatory change on muscle pathology. They were clinically characterized by neck flexion limitation and early joint contracture, but no cardiac problem had developed yet.

**Conclusion:**

Muscle pathology remains helpful in guiding further molecular analyses by direct sequencing of certain genes or by target capture/NGS as a second-tier diagnostic tool, and is crucial for establishing the genotype-phenotype correlation. We also determined the frequencies of the different types of CMD in our cohort which is important for the development of a specific care system for each disease.

## Introduction

Congenital muscular dystrophy (CMD) is a group of genetically and clinically heterogeneous hereditary muscle diseases characterized by early-onset hypotonia and muscle weakness associated with dystrophic change on muscle pathology. The current classification of CMD consists of three major categories: Ullrich type CMD (collagen VI-related dystrophy), merosin-deficient CMD (*LAMA2*-related dystrophy) and CMD with glycosylation defect in alpha-dystroglycan (alpha-dystroglycanopathy); as well as other minor subgroups, such as *LMNA*-related CMD (L-CMD), megaconial type CMD, CMD with integrin alpha-7 defect, and CMD without genetic diagnosis [1].

The incidence of each type of CMD varies among different ethnic populations. Fukuyama type CMD (FCMD), with defective glycosylation of alpha-dystroglycan, is the most common type of CMD in Japan due to a 3-kb insertion founder mutation in the *FKTN* 3’ untranslated region [2]. Merosin-deficient CMD (MDCMD) accounts for around 40% of CMD patients in European countries [3], but is relatively uncommon in Asia. By contrast, the incidence of Ullrich type CMD (UCMD) is similar among different populations [3–5].

In addition to early-onset hypotonia and weakness, each type of CMD has its specific clinical manifestations. Patients with UCMD typically present with distal joint hyperlaxity and early proximal joint contracture. Associated features including developmental dysplasia of the hip (DDH), torticollis and keloid are also often observed. As mentioned above, UCMD is often caused by heterozygous *de novo* mutation in one of the *COL6A1*, *COL6A2*, and *COL6A3* genes, encoding alpha-1, 2 and 3 chains of collagen VI, respectively [1, 6]. These three collagen VI chains assemble to form extracellular matrix [7]. MDCMD is inherited with autosomal recessive trait and characterized by floppiness in infancy, early joint contracture and cerebral white matter lesion. MDCMD is caused by mutations in the *LAMA2* gene, which encodes the human laminin α2 chain (merosin) to form the long-arm coiled coil around skeletal muscle fibers[7]. The characteristic phenotype of MDCMD includes congenital hypotonia, no acquisition of walking ability and white matter signal abnormalities on brain MRI [8]. CMD with defective glycosylation of alpha-dystroglycan is a disease entity involving several enzymes with clinical syndromes ranging from the most severe Walker-Warburg syndrome (WWS) to milder hypotonia without congenital anomaly of the eye or the brain [9]. To date, more than 17 genes have been found to be responsible for this disease [10–17]. *LMNA* mutations have been associated with variable diseases including Emery-Dreifuss muscular dystrophy (EDMD), neuropathy, lipodystrophy, and progeria [18]. L-CMD is often characterized clinically by early joint contracture and neck flexion limitation, and pathologically by marked inflammatory change [19, 20]. Cardiomyopathy or conduction defects are not usually present at early stage. Of note, almost all these causative genes of CMD could also result in a milder phenotype mimicking limb-girdle muscular dystrophy.

In this study, we report the detailed phenotype and genotype of Taiwanese patients with CMD, as confirmed by various mutation analyses, including target capture/next-generation sequencing (NGS).

## Patients and methods

### Patients

A total of 48 patients with a suspected diagnosis of CMD at Kaohsiung Medical University Hospital (KMUH) between 1996 and 2016 were enrolled. All had muscle biopsy analyzed at KMUH. The diagnosis of CMD was based on early-onset of hypotonia and proximal muscle weakness (age of onset < 2 years) with dystrophic change on muscle pathology defined as necrotic and regenerating process together with endomysial fibrosis. The following histochemistry and immunohistochemistry studies and genetic analyses were performed based on diagnostic purpose. Genomic DNA was extracted from whole blood using the Puregene DNA Isolation Kit (Gentra, Minneapolis, MN, USA) according to the manufacturer’s instructions. This study was approved by the institutional review board of Kaohsiung Medical University Hospital. Written informed consent was obtained from all participants undergoing molecular analysis.

### Histochemistry

Biopsied muscle specimens were snap-frozen in isopentane cooled in liquid nitrogen. A serial frozen section was stained by a battery of histochemical methods including hematoxylin and eosin (H&E), modified Gomori-trichrome (mGt) and NADH-tetrazolium reductase (NADH-TR).

### Immunohistochemistry (IHC)

Muscle biopsy cryosections of 6 μm thickness were immunostained with commercially available antibodies against collagen VI (Biomedicals, USA), alpha-dystroglycan (VIA4) (Upstate Biotechnology, USA) and laminin α2 (5H2) (Chemicon, USA) according to the standard protocols with a Ventana Benchmark automated stainer [21].

### Next-generation sequencing (NGS) analysis

The capture probe library contains 247 genes related to a broad spectrum of neuromuscular diseases (NMDs) [22], such as congenital myopathy, congenital muscular dystrophy, congenital myasthenic syndrome, motor neuron disease, arthrogryposis multiplex congenita, and other myopathies.[23] Among them, 25 genes (S1 Table) are known to be responsible for CMD. All coding exons and at least 20 bp of flanking intronic sequences of target genes were captured and sequenced on Illumina HiSeq2000. Sequence alignment, analytical pipeline and variant calling have been previously published [22].

### DHPLC analysis

Equal volumes of the amplified PCR products from a patient and a wild-type (male) control were mixed, denatured at 95°C for 5 minutes, and then incubated at 65°C for 30 minutes to obtain heteroduplexes. Mutational screening, performed for all amplified fragments from each patient, was carried out by DHPLC on a Wave DNA Fragment Analysis System (Transgenomic Inc., San Jose, CA, USA) using a DNASep column (Transgenomic Inc.). DNA molecules eluted from the column were detected by scanning with a UV detector at 260 nm. For DHPLC analysis, heterozygous profiles were identified by visual inspection of the chromatograms [24]. The PCR information for DHPLC analysis was available in S2 Table.

### RT-PCR analysis

First strand cDNA synthesis was performed using Advantage RT-for-PCR Kit (Clontech, Mountain View, CA, USA). Briefly, the reaction mixture, containing 1 ug of total RNA, 1 uM of oligo(dT)_18_, 500 uM of each dNTP, 20 units of RNase inhibitor, 200 units of MMLV reverse transcriptase, and 4 ul of 5x reaction buffer (250 mM Tris-HCl, pH 8.3, 375 mM KCl, 15 mM MgCl_2_), was incubated at 42°C for 1 hr followed by 5 min at 94°C. PCR amplifications of *LAMA2* cDNAs were performed using the primers and conditions described by [25]. The 12 overlapping primer sets cover most (>99%) of the coding sequence of *LAMA2* mRNA.

## Results

**Fig 1:**
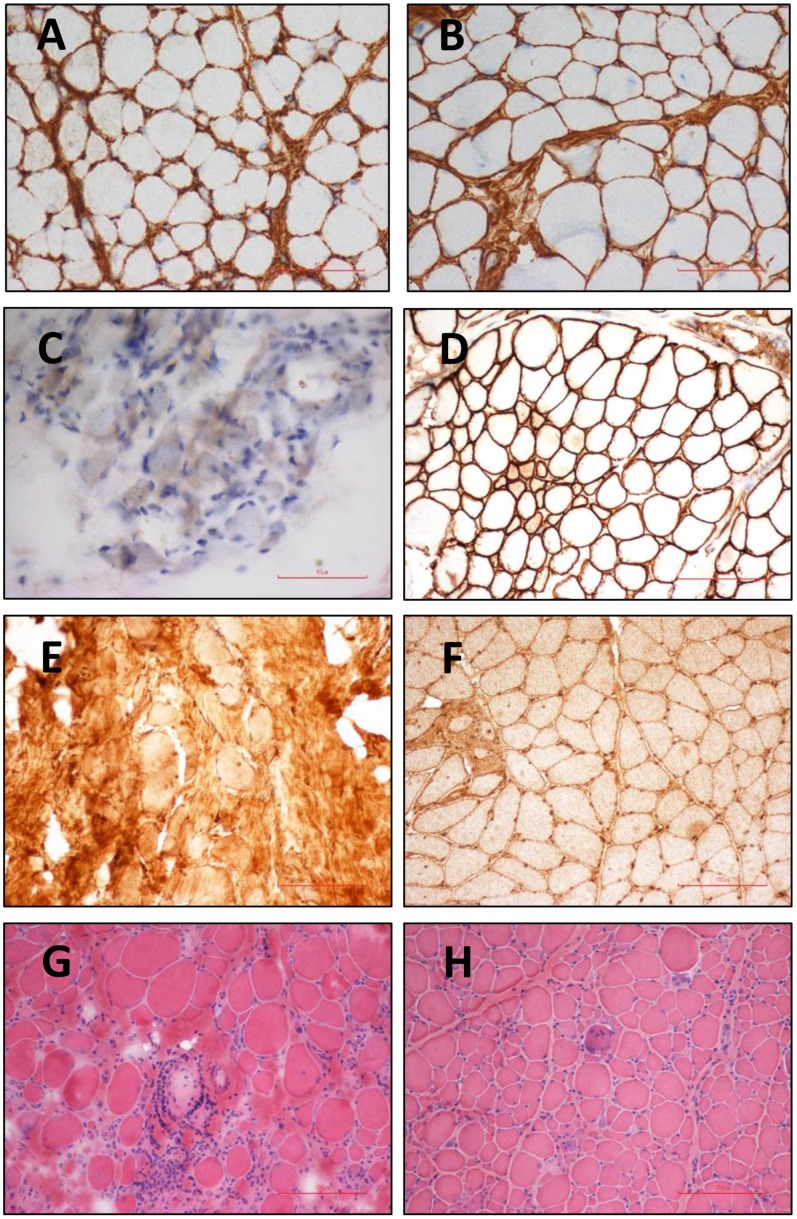
Histochemistry and immunohistochemistry. Sarcolemma specific deficiency of collagen VI was observed in skeletal muscle sample of patent 14 with UCMD (A) compared with the intact staining of collagen VI in control sample (B) Merosin staining was absent in skeletal muscle sample of patient 21 with MDCMD (C) while the staining is well preserved in control sample (D) Alpha-dystroglycan staining was very faint in the patient with glycosylation defect (E) in contrast with the intact staining in control muscle sample (F) Marked inflammatory cell infiltration in some fascicles (G) in addition to dystrophic changes with fibrosis (H) was seen in the patient with L-CMD.

By using histochemistry and IHC studies, we identified 17 patients with sarcolemma-specific collagen VI deficiency (SSCD), 6 patients with partial/complete merosin deficiency, two with reduced alpha-dystroglycan staining, and two with marked inflammatory change in addition to dystrophic change on muscle pathology (Fig 1A~1H). In the following, we describe the detailed phenotype and genotype of each type of CMD. Among the remaining 21 patients with intact IHC staining on muscle pathology, five had NGS analysis but no known mutation has been identified, and the other 16 received no further molecular analysis.

### UCMD

**Fig 2:**
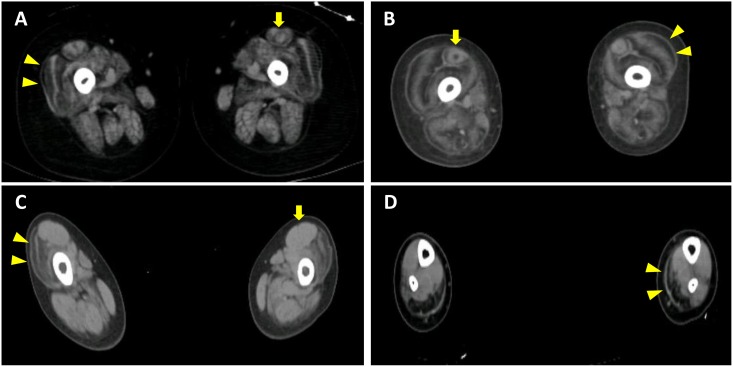
Muscle CT findings. For UCMD, the periphery of vastus lateralis was predominantly affected (arrowhead) with variable involvement of rectus femoris (arrow) (A from patient 7 and B from patient 8). For L-CMD, vastus lateralis was preferentially involved with hypertrophy of rectus femoris at thigh level (C), and medial head of gastrocnemius was predominantly affected at calf level (D).

Fifteen of the 17 patients with SSCD received further mutation analysis using NGS due to the large number of exons of the *COL6A* genes. The phenotype and genotype of these 15 patients were summarized in Table 1. All 15 patients exhibited mild hypotonia in their first years and distal hyper-extensibility in addition to proximal muscle weakness. Proximal joint contracture was observed in 9 patients at the first visit. Two have died due to respiratory failure. All had normal intelligence and cardiac function. Spine deformity was observed in all patients older than 10 years old even though some of them are still ambulant. Thirteen patients were able to reach the milestone of independent walking although 5 in 13 deteriorated later and became confined to a wheelchair. The CK levels ranged from 200 to 600 IU/L. Muscle CT showed preferential involvement in the peripheral part of vastus lateralis and central part of rectus femoris clearly in 5 patients and equivocally in 4 patients of the 13 patients whose muscle images were available for analysis (Fig 2A and 2B).

**Table 1:** Summary of the patients with UCMD.

	Sex	Age (Y)	Proximal joint contracture	Distal hyperlaxity	Keloid	Scoliosis	DDH	Torticollis	Walk independently	Loss of ambulation (Y)	Pathogenic variants in the *COL6A* genes (all heterozygous unless otherwise indicated)
P1	M	17#	p	p	?	p	n	n	Yes (>2y)	7y	COL6A1: c.850 G>A (p.Gly284Arg)
P2	M	22	p	p	n	p	p	n	Yes (1y6-7m)	12y	COL6A1: c.815 G>T (p.Gly272Val)
P3	F	22	p	p	n	p	n	n	Yes (1y1-2m)	not yet	COL6A1: c.868 G>A (p.Gly290Arg)
P4	F	15	p	p	p	p	n	n	Yes (<2y)	not yet	COL6A1: c.868 G>A (p.Gly290Arg)
P5^	F	14	n	p	p	p	p	p	Yes (1y2m)	not yet	COL6A3: c.1676_1677insT (p.Lys560*) (homo)
P6^	M	13	n	p	n	p	n	n	Yes (1y2m)	not yet	COL6A3: c.1676_1677insT (p.Lys560*) (homo)
P7	M	10	p	p	p	n	n	n	Yes (1y6-7m)	8y	COL6A1: c.815 G>T (p.Gly272Val)
P8	M	17	p	p	n	p	n	n	Yes (1y6m)	10y	COL6A2: c.955-2A>G
P9	F	14	p	p	p	p	n	n	Yes (1y2m)	not yet	COL6A1: c.868 G>A (p.Gly290Arg)
P10	M	6	n	p	Equivocal	n	n	n	Yes (1y2m)	not yet	Not found
P11	M	14#	p	p	?	p	n	n	Yes (<2y)	12y	COL6A3: c.6309+2 T>A
P12	M	7	n	p	p	n	n	n	Yes (1y6m)	not yet	COL6A3: c.6157G>T (p.Gly2053Cys)
P13	M	6	p	p	p	n	p	p	Yes (2y)	not yet	COL6A1: c.886G>A (p.Gly296Arg)
P14	M	1y11m	n	p	Equivocal	p	p	n	no	no	COL6A2: c.1043_1051delCTGGAAA, (p.Pro348_Asn350del)
P15	M	5y1m	p	p	p	n	n	n	no	no	COL6A2: exon5 deletion

^#^: the age of death;

^: siblings;

^?^: no record;

p: present; n: nil

Seven patients harbored *de novo* heterozygous missense mutations in *COL6A1*; all mutations have been reported and located in the triple helix domain. The most frequent mutation was c.868G>A, p.Gly290Arg in 3 patients, followed by c.815G>T, p.Gly272Val in 2 patients. Likely pathogenic variants in *COL6A2* were identified in 3 patients. Patient 8 carried a variant at the consensus splicing site (c.955-2A>G), patient 14 carried an out-of-frame deletion, c.1043_1051delCTGGAAACC, p.Pro348_Asn350del (in-frame), and patient 16 had exon 5 deletion, most likely all leading to a truncated protein. As for *COL6A3*, patient 5 and 6 who are siblings, each harbored a homozygous c.1676_1677insT, p.Lys560* frame shift variant resulting in truncated protein. A c.6309+2T>A in *COL6A3* was identified in patient 11. Patient 15 harbored a c.6157G>T, p.Gly2053Cys missense variant that has been reported in collagen-VI related myopathy [26].

Probable pathogenic variants in *COL6A* genes were not identified in Patient 10 except for the presumably benign variant, c.2503G>A (p.Val835Ile) in *COL6A2* which has been reported as a SNP with allele frequency of 0.008.

### MDCMD

Six patients from 5 families were diagnosed with MDCMD by IHC in this study and received further mutation analysis. The phenotype and genotype of these 6 patients were summarized in Table 2. All 6 patients showed floppiness by the age of 6 months and marked motor development delay. The CK levels ranged from 1,500 to 7,000 IU/L. Only one was able to walk without assistance for some while. Brain MRI identified typical white matter hyperintensity in all patients. Three patients developed epilepsy and had mild to moderate mental retardation, as assessed by Wechsler Intelligence Scale for Children (WISC). All patients developed marked spine deformity and joint contracture by the age of 10 years, and five patients required respiratory support starting from their teens. Only one patient had mild left ventricle dilatation and valve regurgitation.

**Table 2:** Summary of the patients with MDCMD.

	Sex	Current age/ Age of onset	Hypotonia in infancy	Walk independently	Epilepsy	Intelligence	Brain MRI (abnormal white matter signal)	Pathogenic variants in *LAMA2*
P16	F	31y/6m	p	n	p	mild MR	p	c.624 delC (p.Leu209*) (m)
								c.2209-3_2209–2 delCA (f)
P17^	M	27y/6m	p	n	p	moderate MR	p	c.8654 T>C (p.Leu2885Pro) (m)
								c.2945 insG (p.Ser982Arg fs*16) (f)
P18^	M	#12y/4m	p	n	p	mild MR	p	c.8654 T>C (p.Leu2885Pro) (m)
								c.2945 insG (p.Ser982Arg fs*16) (f)
P19	M	18y/4m	P	n	n	borderline	p	c.6513_6515 delTGT (p.Val2172del) (m)
								c.4311 G>A (p.Gln437Gln) (f)
P20	F	16y/4m	P	p	n	normal	p	c.8989-12 C>G (m)
								c.2451+6 A>G (f)
P21	F	18y/5m	p	n	n	normal	p	c.2049_2050 delAG (p.Arg683Ser fs*21) (m)
								c.1303 C>T (p.Arg435*)

^#^: the age of death;

^: siblings;

p: present; n: nil; f: father; m: mother

Ten different sequence variants in *LAMA2* were identified in all 6 patients using DHPLC followed by direct sequencing for confirmation. As Patients 17 and 18 were siblings, they carried the same two variants. All these variants were inherited from their parents except for the nonsense mutation (c.1303C>T, p.Arg435*) in patient 21. Three frameshift deletions or insertions (c.624 delC, c.2049_2050delAG, c.2945insG), four splice site variants (c.2209-3_2209-2delCA, c.2451+6A>G, c.4311G>A, c.8989-12 C>G), and one nonsense mutation (c.1303C>T, p.Arg435*) were expected to produce truncated proteins. One in-frame deletion (c.6513_6515delTGT) and one missense mutation (c.8654T>C, p.Leu2885Pro) might lead to partial protein expression. The c.2049_2050delAG and c.6513_6515delTGT mutations have previously been reported [27], while other mutations have not been reported before. None of the newly identified mutations were found in a scan of 100 normal individuals. It appears that the variants are widely distributed and have no hotspot region in the *LAMA2* gene.

**Fig 3:**
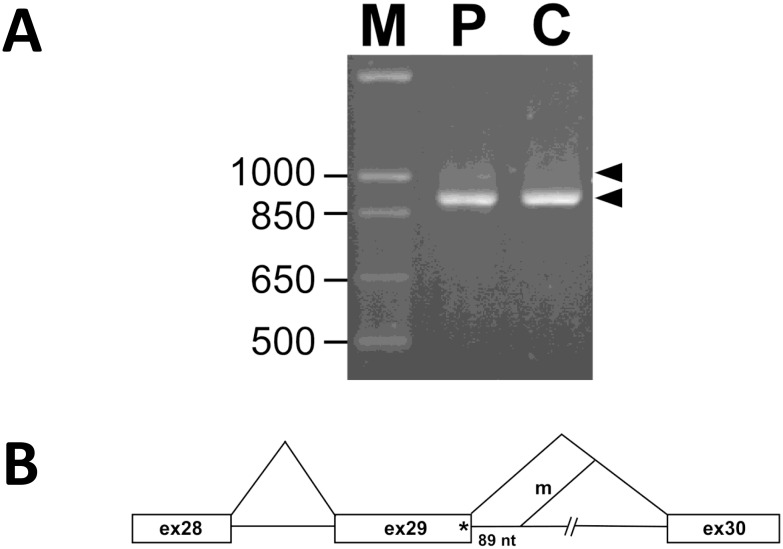
cDNA analysis for patient 19 to elucidate the pathogenicity of c.4311 G>A in *LAMA2*. (A) RT-PCR analysis of *LAMA2* mRNA of patient 4 and control individual. (B) Aberrant splicing of the mutant *LAMA2* mRNA in patient 4.

The synonymous change, c.4311G>A, p.Gln1437Gln identified in patient 19 is located at the last nucleotide of exon 29, and 5 splice prediction algorithms predict this to be deleterious. Further cDNA analysis using primers located at exon 26 and exon 33 revealed a normal cDNA product of 911 bp in the control individual, while an aberrant product approximately 1,000 bp in addition to the normal 911 bp band was observed in patient 4 (Fig 3A). Sequence analysis of the aberrant DNA fragment showed that the mutation abolished the original 5’ splice donor site of intron 29 and a cryptic site 89 bp downstream within the intron was used instead (Fig 3B). Our results clearly demonstrate that the c.4311G>A mutation is a splice site mutation.

### CMD with glycosylation defect in alpha-dystroglycan

In our cohort, reduced alpha-dystroglycan staining on IHC was found in two patients. The first patient, a currently 16 year-old-girl showed floppiness and delayed motor development in her infancy and the best motor function she could attain was walking with support. In addition to progressive proximal muscle weakness, she also presented with myopathic face, pigeon chest and moderate mental retardation (IQ 40, assessed by WISC). Neither white matter lesion nor other structural abnormality was found on brain MRI. The CK level was 2,081 IU/L when she was referred to our hospital at age 10. Lung function test showed rapid deterioration in the following 4 years (FVC: 46.4% decreased to 23%) and she has now started non-invasive ventilation at night. Electrocardiogram and cardiac echo revealed normal findings at age 12; scoliosis gradually developed after age 10.

Compound heterozygous mutations in *POMT1*, encoding protein O-mannosyl transferase 1 for carrying out glycosylation on alpha-dystroglycan, were later identified by mutation analysis and confirmed by parental study. The analysis was performed using NGS on account of the considerable number of causative genes for alpha-dystroglycanopathy. The c.793C>T, p.Arg265* is a nonsense mutation causing premature termination of translation; the other mutation, c.1859G>C (p.Arg620Pro) has never been reported but computer-based algorithms (SIFT and Polyphen2) have predicted it to be damaging. Taking the clinical and pathological findings together, the mutation is most likely pathogenic.

Surprisingly, the other patient with mildly reduced alpha-dystroglycan staining on IHC was found to have compound heterozygous mutations in *TRAPPC11* which has previously been reported to be responsible for LGMD2S [28]. This patient presented with infantile-onset cataracts and fatty liver in addition to proximal muscle weakness. The detailed information of this patient has been published recently [29].

### L-CMD

Two sisters, aged 5 and 7 respectively, were clinically diagnosed with CMD. Their parents denied consanguinity. All IHC showed intact staining and routine histochemistry revealed diffuse dystrophic changes with marked lymphocyte infiltration in only some fascicles. Both patients displayed bizarre gait ever since they started walking independently at around age 1.5, with frequent falls. The parents refused to acknowledge the floppiness during their infancy. Progressive proximal weakness developed thereafter and the patients lost the ability to walk at age 4 and 6, respectively. Physical examination showed markedly limited neck flexion and joint contracture in elbow, hip and ankle. No cardiac involvement but mild ventilatory defect was found. The CK level was around 600 IU/L. Taking clinical and pathological findings together, L-CMD was highly suspected. At the thigh level of muscle CT, vastus lateralis showed similar involvement at the periphery to that seen in UCMD, but rectus femoris was usually spared and hypertrophic (Fig 2C). At the calf level, the medial head of the gastrocnemius was preferentially affected (Fig 2D).

**Fig 4:**
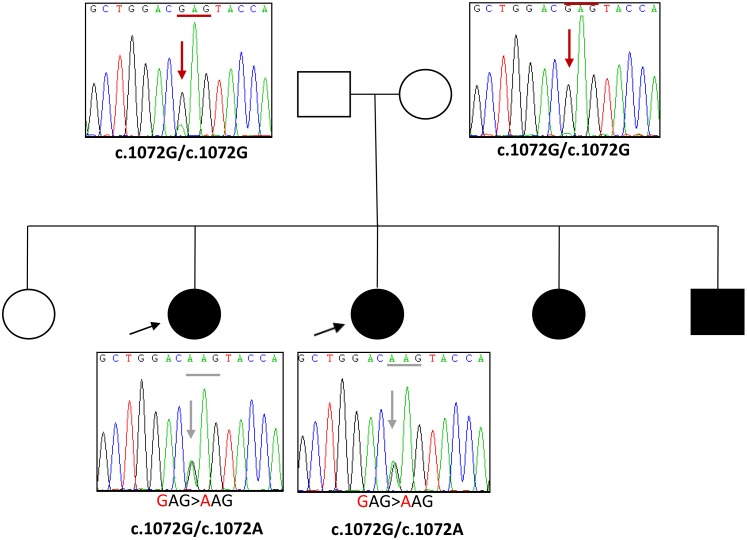
Pedigree and sequencing results of the patients with L-CMD.

Direct sequencing identified a heterozygous mutation in *LMNA*, c.1072G>A, p.Glu358Lys in these two patients (Fig 4), which has been reported as a frequent mutation in L-CMD [20]. Their parents were both asymptomatic and did not carry the same mutation. However, in addition to these two patients, there were two other symptomatic children from the same family who were later confirmed to carry the same mutation too. These results highly suggest that one of the parents may have germline mosaicism.

## Discussion

The target gene capture/deep sequencing approach can greatly facilitate the pace of genetic diagnosis of muscle diseases. Confirmed molecular diagnoses of muscle diseases can assist in genetic counseling and carrier detection as well as guide therapeutic options for individualized treatment in the future. However, conclusively proving the pathogenic variant is the most difficult part after obtaining the NGS results, therefore, establishment of genotype-phenotype correlation is crucial in making the final diagnosis. Detailed clinical and pathological investigation remains the basis [22, 27, 29].

Our results suggest that the most common CMD in Taiwan might be the Ullrich type. Collagen VI-related dystrophy is known to encompass a clinical continuum with UCMD and Bethlem myopathy (BM) at each end of the spectrum. Intermediate phenotypes are not yet well defined and the same mutation could cause either UCMD or BM [30]. Therefore, collagen VI-related myopathy or VI-related dystrophy is now preferentially referred. In our cohort, all patients are early-onset; two were categorized as “early severe”; six were “moderate progressive”; the remaining 7 patients were “mild” according to phenotypic stratification [20, 31]. The proportion of “early severe” group is around 13.3% which is much less than the previously reported 19.4–25.7% [31, 32]. As “early severe” patients might present with arthrogryposis multiplex congenita, they might not have been recognized as CMD and referred to our center. Nevertheless, proximal muscle weakness with distal hyperlaxity is characteristic for UCMD, especially together with proximal joint contracture. The muscle CT findings of our patients did not well correlate with the clinical severity especially in very young children, probably due to early assessment and small muscle mass. Although muscle MRI has been reported to be helpful for the diagnosis of collagen VI-related myopathy [33], some difficulty remains arranging muscle MRI in Taiwan, due to availability and cost. In addition, patients have to wait for months for MRI and the children might need more sedation for it. However, the muscle CT performed on our patients showed similar pattern to MRI, suggesting the possible compatibility between these two imaging methods.

In our cohort, a homozygous mutation in *COL6A3*, c.1676_1677insT, p.Lys560* was identified in patients 6 and 7, who are siblings. Both asymptomatic parents are heterozygous for this variant. Homozygous mutation usually results in complete loss of protein and more severe phenotype; however, the IHC staining on skeletal muscles from these two patients showed only partial deficiency of collagen VI and their clinical phenotype was not “early severe”. Tracing back to their family history, one paternal aunt also had similar symptoms and the mutation screening showed that she carried the same homozygous mutation, hinting that both of her parents (grandparents of patients 6 and 7) might be heterozygous and therefore most likely also passed the variant to the father of patients 6 and 7. However, as their DNA samples were not available, we could not confirm whether they were both carriers or not. Although the patients’ parents denied consanguinity, further linkage analysis may be helpful to clarify the haplotype of this family.

MDCMD is the most common CMD in many European countries [3]. In Asia, the largest series with 43 patients has been reported from China [34] but no clear epidemiology has been described in either China or other Asian countries. In our cohort, it accounts for the second most frequent CMD in Taiwan, following UCMD. No recurrent mutation has been identified in this study. The phenotype of our patients is relatively homogeneous, consistent with previous reports. It is worth noting that the proportion of our patients with mental retardation and epilepsy seems to be larger than in previous description (50% versus around 15%) [35]. The mutations identified here are distributed throughout the gene and there seems to be no clear genotype-phenotype correlation. However, more patients should be studied for further clarification.

In our series, only one patient was identified to have compound heterozygous mutations in one of the alpha-dystroglycanopathy related genes, *POMT1*. Cardiomyopathy and respiratory failure could occur in patients with *POMT1* mutations but are uncommon [36]. Our patient did not show cardiac involvement but severe restrictive respiratory defect had already occurred by age 12. More patients should be studied to understand the natural course of Taiwanese patients. Interestingly, the incidence of CMD with glycosylation defect in alpha-dystroglycan has been increasing in European countries and Australia in recent years [5, 37]. As our patients were enrolled based on clinical and muscle pathology findings, there might be a selective bias as some floppy babies with congenital brain/eye anomalies might not be recognized as CMD or could not be referred for muscle biopsy due to critical clinical condition.

One of our patients with decreased staining of alpha-dystroglycan was identified to have *TRAPPC11* mutations by NGS. This implied that the defect of TRAPPC11 might result in the perturbation of glycosylation occurring in the endoplasmic reticulum (ER) or the Golgi apparatus as TRAPPC11 is important for TRAPPC complex assembly and membrane traffic specifically between the ER and ER-to-Golgi intermediate compartment [28, 38]. Further studies are necessary to clarify the relationship among TRAPPC11, glycosylation and ER-to-Golgi trafficking system.

The clinical, pathological and imaging features of patients with L-CMD presented in this study are similar to previous reports of L-CMD except for head drop [19, 20]. Our patients showed limited neck flexion reflecting rigid spine, one of classic EDMD phenotypes. As cardiac involvement might not develop in the early stage, either head drop or cervical contracture leading to neck flexion limitation together with inflammatory change on muscle pathology is truly characteristic for L-CMD. In this family, germline mutation of one parent was suspected as only one heterozygous mutation was identified and multiple children were symptomatic but both parents were asymptomatic. A broader variety of tissue samples from the parents could be tested to confirm the mosaicism [39].

In our cohort, we did observe that the CK level moved in different ranges for different CMD subtypes. It was the highest in the patient with *TRAPPC11* mutations which could be up to 10,000 IU/L. In the patients with MDCMD and glycosylation defect with *POMT1* mutations, the CK level ranged in the thousands but did not reach 10,000 IU/L. The CK levels in both L-CMD and UCMD were only mildly elevated, rarely over 1,000 IU/L. Taken together with clinical and pathological features, the CK level might also be helpful for differentiating the subtypes of CMD.

In conclusion, we have defined the frequencies of different types of CMD in a large patient cohort and demonstrated the utility of histochemistry/IHC in categorization to further guide molecular analyses. Molecular studies, with comprehensive target capture/NGS as a second-tier, can confirm the disease subtype and provide accurate genetic counseling for the family, while facilitating further studies for the understanding of the genetic basis and phenotypic variability of the CMDs. Results presented here are also important for the establishment of the national registry of patients with CMD in Taiwan.

## Supporting information

S1 Table Congenital muscular dystrophy related genes in the capture probe library. (DOCX)

S2 Table PCR primer sequences, PCR product sizes, PCR conditions and DHPLC analysis conditions for heteroduplex analysis. (DOCX)
