# Correction: Comprehensive target capture/next-generation sequencing as a second-tier diagnostic approach for congenital muscular dystrophy in Taiwan

**DOI:** 10.1371/journal.pone.0183406

**Published:** 2017-08-10

**Authors:** Wen-Chen Liang, Xia Tian, Chung-Yee Yuo, Wan-Zi Chen, Tsu-Min Kan, Yi-Ning Su, Ichizo Nishino, Lee-Jun C. Wong, Yuh-Jyh Jong

In Table 1, the COL6A2 mutation in Patient 14 is listed incorrectly. The mutation should be: c.1043_1051delCTGGAAACC, (p.Pro348_Asn350del). Please see the corrected Table 1 here.

**Table 1:** Summary of the patients with UCMD.

	Sex	Age (Y)	Proximal joint contracture	Distal hyperlaxity	Keloid	Scoliosis	DDH	Torticollis	Walk independently	Loss of ambulation (Y)	Pathogenic variants in the *COL6A* genes (all heterozygous unless otherwise indicated)
P1	M	17#	p	p	?	p	n	n	Yes (>2y)	7y	COL6A1: c.850 G>A (p.Gly284Arg)
P2	M	22	p	p	n	p	p	n	Yes (1y6-7m)	12y	COL6A1: c.815 G>T (p.Gly272Val)
P3	F	22	p	p	n	p	n	n	Yes (1y1-2m)	not yet	COL6A1: c.868 G>A (p.Gly290Arg)
P4	F	15	p	p	p	p	n	n	Yes (<2y)	not yet	COL6A1: c.868 G>A (p.Gly290Arg)
P5^	F	14	n	p	p	p	p	p	Yes (1y2m)	not yet	COL6A3: c.1676_1677insT (p.Lys560*) (homo)
P6^	M	13	n	p	n	p	n	n	Yes (1y2m)	not yet	COL6A3: c.1676_1677insT (p.Lys560*) (homo)
P7	M	10	p	p	p	n	n	n	Yes (1y6-7m)	8y	COL6A1: c.815 G>T (p.Gly272Val)
P8	M	17	p	p	n	p	n	n	Yes (1y6m)	10y	COL6A2: c.955-2A>G
P9	F	14	p	p	p	p	n	n	Yes (1y2m)	not yet	COL6A1: c.868 G>A (p.Gly290Arg)
P10	M	6	n	p	Equivocal	n	n	n	Yes (1y2m)	not yet	Not found
P11	M	14#	p	p	?	p	n	n	Yes (<2y)	12y	COL6A3: c.6309+2 T>A
P12	M	7	n	p	p	n	n	n	Yes (1y6m)	not yet	COL6A3: c.6157G>T (p.Gly2053Cys)
P13	M	6	p	p	p	n	p	p	Yes (2y)	not yet	COL6A1: c.886G>A (p.Gly296Arg)
P14	M	1y11m	n	p	Equivocal	p	p	n	no	no	COL6A2: c.1043_1051delCTGGAAACC, (p.Pro348_Asn350del)
P15	M	5y1m	p	p	p	n	n	n	no	no	COL6A2: exon5 deletion

#: the age of death;

^: siblings;

?: no record;

p: present; n: nil

In Table 2, the LAMA2 mutation in Patient 20 is listed incorrectly. The mutation should be: c.2451-6 A>G. Please see the corrected Table 2 here.

**Table 2:** Summary of the patients with MDCMD.

	Sex	Current age/ Age of onset	Hypotonia in infancy	Walk independently	Epilepsy	Intelligence	Brain MRI (abnormal white matter signal)	Pathogenic variants in *LAMA2*
P16	F	31y/6m	p	n	p	mild MR	p	c.624 delC (p.Leu209*) (m)
								c.2209-3_2209–2 delCA (f)
P17^	M	27y/6m	p	n	p	moderate MR	p	c.8654 T>C (p.Leu2885Pro) (m)
								c.2945 insG (p.Ser982Arg fs*16) (f)
P18^	M	#12y/4m	p	n	p	mild MR	p	c.8654 T>C (p.Leu2885Pro) (m)
								c.2945 insG (p.Ser982Arg fs*16) (f)
P19	M	18y/4m	P	n	n	borderline	p	c.6513_6515 delTGT (p.Val2172del) (m)
								c.4311 G>A (p.Gln437Gln) (f)
P20	F	16y/4m	P	p	n	normal	p	c.8989-12 C>G (m)
								c.2451-6 A>G (f)
P21	F	18y/5m	p	n	n	normal	p	c.2049_2050 delAG (p.Arg683Ser fs*21) (m)
								c.1303 C>T (p.Arg435*)

#: the age of death;

^: siblings;

p: present; n: nil; f: father; m: mother

The LAMA2 mutation in Patient 20 is also listed incorrectly in the fourth sentence of the second paragraph of the MDCMD subsection of the Results section. The correct sentence is: Three frameshift deletions or insertions (c.624 delC, c.2049_2050delAG, c.2945insG), four splice site variants (c.2209-3_2209-2delCA, c.2451-6A>G, c.4311G>A, c.8989-12 C>G), and one nonsense mutation (c.1303C>T, p.Arg435) were expected to produce truncated proteins.

## References

[1] Liang W-C, Tian X, Yuo C-Y, Chen W-Z, Kan T-M, Su Y-N, et al (2017) Comprehensive target capture/next-generation sequencing as a second-tier diagnostic approach for congenital muscular dystrophy in Taiwan. PLoS ONE 12(2): e0170517 https://doi.org/10.1371/journal.pone.0170517 28182637 10.1371/journal.pone.0170517 PMC5300266

