# Supplementary material for: Comprehensive target capture/next-generation sequencing as a second-tier diagnostic approach for congenital muscular dystrophy in Taiwan
Source: PLoS One. 2017 Feb 9;12(2):e0170517. doi: 10.1371/journal.pone.0170517 (PMC5300266; doi:10.1371/journal.pone.0170517)
Supplement: S1 Table — (DOCX) [file pone.0170517.s001.docx]

**S1 Table.** Congenital muscular dystrophy related genes in the capture probe library

| Group2.NMD_CMD (Congenital Muscular Dystrophy) | gene | NM# | inheritance | OMIM |
| --- | --- | --- | --- | --- |
| 1 | ALG13 | NM_001099922.2 | X-Linked | 300776 |
| 2 | B3GALNT2 | NM_152490.4 | AR | 610194 |
| 3 | CHKB | NM_005198.4 | AR | 612395 |
| 4 | COL6A1 | NM_001848.2 | AD/AR | 120220 |
| 5 | COL6A2 | NM_001849.3 | AD/AR | 120240 |
| 6 | COL6A3 | NM_004369.3 | AD/AR | 120250 |
| 7 | DNM2 | NM_001005360.2 | AD/AR | 602378 |
| 8 | DPM1 | NM_003859.1 | AR | 603503 |
| 9 | DPM2 | NM_003863.3 | AR | 603564 |
| 10 | FHL1 | NM_001449.4 | X-Linked | 300163 |
| 11 | FKRP | NM_024301.4 | AR | 606596 |
| 12 | FKTN | NM_001079802.1 | AR | 607440 |
| 13 | GMPPB | NM_021971.2 | AR | 615320 |
| 14 | ISPD | NM_001101426.3 | AR | 614631 |
| 15 | ITGA7 | NM_002206.2 | AR | 600536 |
| 16 | LAMA2 | NM_000426.3 | AR | 156225 |
| 17 | LARGE | NM_004737.4 | AR | 603590 |
| 18 | POMGNT1 | NM_017739.3 | AR | 606822 |
| 19 | POMGNT2 | NM_032806.5 | AR | 614828 |
| 20 | POMK | NM_032237.4 | AR | 615247 |
| 21 | POMT1 | NM_007171.3 | AR | 607423 |
| 22 | POMT2 | NM_013382.5 | AR | 607439 |
| 23 | SEPN1 | NM_020451.2 | AR | 606210 |
| 24 | TCAP | NM_003673.3 | AD/AR | 604488 |
| 25 | TMEM5 | NM_014254.2 | AR | 605862 |
