# Supplementary material for: Comprehensive target capture/next-generation sequencing as a second-tier diagnostic approach for congenital muscular dystrophy in Taiwan
Source: PLoS One. 2017 Feb 9;12(2):e0170517. doi: 10.1371/journal.pone.0170517 (PMC5300266; doi:10.1371/journal.pone.0170517)
Supplement: S2 Table — (DOCX) [file pone.0170517.s002.docx]

**S2 Table.** PCR primer sequences, PCR product sizes, PCR conditions and DHPLC analysis conditions for heteroduplex analysis

| **Region** |  | **Primer** | **size** | **Annealing temperature(°C)** | **Oven temperature(°C)** |
| --- | --- | --- | --- | --- | --- |
| EXON 1 | F | ctctggctcccgagaagt | 320 | 50 °C | 63.5/66 |
|  | R | tcatggcattgaacttgaac |  |  |  |
| EXON 2 | F | gggttactttaatgctccgaaa | 352 | 50 °C | 57.5/58.5 |
|  | R | cttggcctcaacatctcaca |  |  |  |
| EXON 3 | F | tcacatgatggttgttttaacc | 315 | 50 °C | 55.5/56.5 |
|  | R | tatcctttcacccacaaacc |  |  |  |
| EXON 4-1 | F | gccatggttgtcaaaacaaa | 371 | 50 °C | 52/59.5 |
|  | R | cccagtgcggggataaata |  |  |  |
| EXON 4-2 | F | cggcctggaaactggatt | 400 | 50 °C | 53.5/57 |
|  | R | tggagatgggagacacatga |  |  |  |
| EXON 5 | F | aattgggagaatgggaagtt | 391 | 50 °C | 52/58 |
|  | R | ccactcctacaaatcactgaaa |  |  |  |
| EXON 6 | F | tgagtcctcacttgtcctca | 325 | 50 °C | 57 |
|  | R | ttacagcgttctctgtcagc |  |  |  |
| EXON 7 | F | ccaggctggctagttttaca | 346 | 53 °C | 55.5/58.5 |
|  | R | gcataaccagccatttcact |  |  |  |
| EXON 8 | F | gacaggaggattgcttgaac | 431 | 50 °C | 53/56.5 |
|  | R | catttttgtatgaaaaagcaaa |  |  |  |
| EXON 9 | F | tctcaataatttgctgctctg | 368 | 50 °C | 53/58 |
|  | R | tggagggaacgctaatacac |  |  |  |
| EXON 10 | F | aaaccctctactctttggtttt | 408 | 50 °C | 57.5/60 |
|  | R | taatgccaaggaaatcaaca |  |  |  |
| EXON 11 | F | tgagagggtaaaatgaagaatg | 400 | 50 °C | 57.5/59 |
|  | R | cagcatttgaagaaaattcca |  |  |  |
| EXON 12 | F | aatttccaaaagtggacacg | 395 | 50 °C | 55.5/62.5 |
|  | R | gttctggcaatccaattca |  |  |  |
| EXON 13 | F | cctatcattcccactccgta | 399 | 50 °C | 57 |
|  | R | gagtggcacaaaaatctcct |  |  |  |
| EXON 14 | F | attgagggtggaggatacaa | 445 | 50 °C | 57.5 |
|  | R | tagggctccgttcttatctg |  |  |  |
| EXON 15 | F | ttctcagcatgcataattgg | 391 | 50 °C | 56/58.5 |
|  | R | aaagccacagcttttggtag |  |  |  |
| EXON 16 | F | cccagtgtcaggaggaatag | 387 | 50 °C | 56/60 |
|  | R | tttaaggtccccagggtagt |  |  |  |
| EXON 17 | F | gcagggagcagactaatgac | 320 | 50 °C | 57.5 |
|  | R | acatgggataaaaggaagtgt |  |  |  |
| EXON 18 | F | gtctctggggtgagaatgac | 311 | 50 °C | 58.5/60 |
|  | R | aggatgatgcaaaggatgat |  |  |  |
| EXON 19 | F | atgtaaacattgccccaact | 357 | 50 °C | 59.5 |
|  | R | tccacactcaagaactcatca |  |  |  |
| EXON 20 | F | aatgcctaacagggcactta | 369 | 50 °C | 55.5/60.5 |
|  | R | taatcccaaaccttcctttt |  |  |  |
| EXON 21 | F | ttgtgcatcttgcttcactt | 356 | 50 °C | 60 |
|  | R | cgttgtatcaatctgtgcttc |  |  |  |
| EXON 22 | F | attgaacagctcctttctga | 412 | 50 °C | 54/57.5 |
|  | R | gaaatgcccaattacagagc |  |  |  |
| EXON 23 | F | tgtgtttaatggaagcatagaaa | 400 | 50 °C | 58/59.5 |
|  | R | aaaactgagcaatggaaacag |  |  |  |
| EXON 24 | F | agagtatgctcccgttatgc | 304 | 50 °C | 61.5 |
|  | R | tgcctgactaaaagacacca |  |  |  |
| EXON 25 | F | gcagatagacatgcagttcg | 387 | 50 °C | 55.5/59.5 |
|  | R | aggccatcaactgacaaaat |  |  |  |
| EXON 26 | F | cttccagaacaatttgaagga | 441 | 50 °C | 55/57.5 |
|  | R | cgaggatcatccccataata |  |  |  |
| EXON 27 | F | caaggcatgaaattgaaatg | 384 | 50 °C | 54.5/55.5 |
|  | R | agcctccatggtttatcttct |  |  |  |
| EXON 28 | F | ttgaggttgacaaaaacgtg | 370 | 50 °C | 56/58 |
|  | R | ctcttgccactgtgtctcac |  |  |  |
| EXON 29 | F | gcgtttgtaagtgatgttgc | 378 | 50 °C | 60.5 |
|  | R | cacacagttgcattccctta |  |  |  |
| EXON 30 | F | tgtgttcatagacacacattca | 300 | 50 °C | 56.5/58.5 |
|  | R | caaatcatagacagcccatgt |  |  |  |
| EXON 31 | F | tttcattgtgacgtcctagc | 400 | 50 °C | 53.5/58.5 |
|  | R | agtggttttgaaagcattgt |  |  |  |
| EXON 32 | F | ttgcatcaaaacaaaagacc | 342 | 50 °C | 62.5 |
|  | R | tggaaatgagacagaactgg |  |  |  |
| EXON 33 | F | ctcaaccatcatccatttga | 382 | 50 °C | 59.5/61.5 |
|  | R | accaattacagccaataccg |  |  |  |
| EXON 34 | F | atttggcatgacactcaatg | 371 | 50 °C | 56/60 |
|  | R | tgggaattaggaagaaggtg |  |  |  |
| EXON 35 | F | ggcatcatttgcaatacatc | 341 | 50 °C | 56.5/59.5 |
|  | R | gaaattccagtcctgtgtca |  |  |  |
| EXON 36 | F | tcacggcaaaatactcttca | 397 | 50 °C | 53.5/56.5 |
|  | R | atctccaggttggaaaaaca |  |  |  |
| EXON 37 | F | caataaaccctaaggcagtga | 354 | 50 °C | 58.5 |
|  | R | tgaatagtccagccatgttc |  |  |  |
| EXON 38 | F | aacagctcaggaaagtcagg | 354 | 50 °C | 55.5/57.5 |
|  | R | ccaatagataggcatggtca |  |  |  |
| EXON 39 | F | aaaagagaggaggcttgaat | 363 | 50 °C | 55.5/57.5 |
|  | R | ctatccacagaaaggccata |  |  |  |
| EXON 40 | F | gatcatttggaagccagatt | 317 | 50 °C | 57.5 |
|  | R | ctgggattttccagactgac |  |  |  |
| EXON 41 | F | gtaccaactgccttctggat | 300 | 50 °C | 54/57.5 |
|  | R | agaatcatcacaaaaatcatgc |  |  |  |
| EXON 42 | F | ccaagtataagctcagggatg | 391 | 50 °C | 53.5/56 |
|  | R | ctggctaaaggcaataaaaga |  |  |  |
| EXON 43 | F | aagttcagccttttcccttt | 397 | 50 °C | 56/59.5 |
|  | R | ttccaactcgagcatttaca |  |  |  |
| EXON 44 | F | cagttaaaatggggtgcatt | 391 | 50 °C | 52/55 |
|  | R | cgcctgaccaaactttttat |  |  |  |
| EXON 45 | F | catttgccatcacacatttt | 389 | 50 °C | 56/57.5 |
|  | R | gaatcctggggaaagacatt |  |  |  |
| EXON 46 | F | atgatggctttgtggttgta | 335 | 50 °C | 55.5 |
|  | R | tagaccatttggggaccata |  |  |  |
| EXON 47 | F | aatggcgcttattgaaaaac | 381 | 46 °C | 54/57.5 |
|  | R | acatacccactcccagacat |  |  |  |
| EXON 48 | F | aaaacaagtctccgcatttt | 373 | 50 °C | 56/58.5 |
|  | R | cgtgatcaagttttacagagc |  |  |  |
| EXON 49 | F | cttttcccctgttgttgact | 440 | 50 °C | Aug-55 |
|  | R | ctttcaaacctgctccacat |  |  |  |
| EXON 50 | F | ggtatttcctgccctacaac | 399 | 46 °C | 56.5/58.5 |
|  | R | atcatcccaggctttttaga |  |  |  |
| EXON 51 | F | agcaatttagccacaagacc | 328 | 50℃ | 56/57 |
|  | R | ccttcctcttgatcaggtaca |  |  |  |
| EXON 52 | F | tgcaagtgcttgagaaagtc | 383 | 46 °C | 54/56 |
|  | R | tttctgcaaccaagggttat |  |  |  |
| EXON 53 | F | gcttttgcatttctttcctt | 394 | 46 °C | 52/59.5 |
|  | R | tcactgctggtgagacaact |  |  |  |
| EXON 54 | F | aaaccagagtttgctgggta | 346 | 50 °C | 57/58.5 |
|  | R | cttgtaggagcagccacac |  |  |  |
| EXON 55 | F | gggtgagtgagatggagaac | 360 | 50 °C | 54/59.5 |
|  | R | gccaagatgagagcagaaat |  |  |  |
| EXON 56 | F | gttctgccagggaatctcta | 330 | 46 °C | 57/58.5 |
|  | R | taggtccacacacacagagg |  |  |  |
| EXON 57 | F | gctatggttgagaggggtga | 394 | 57 °C | 55.5/57 |
|  | R | cctcccctgcatagctgtag |  |  |  |
| EXON 58 | F | aggagggaaacaatgaggat | 379 | 46 °C | 55.5/60 |
|  | R | tcttccttctgaaatgactcag |  |  |  |
| EXON 59 | F | acgcactgagcactcaataa | 450 | 46 °C | 54/57 |
|  | R | cctcaggctcagacaaataaa |  |  |  |
| EXON 60 | F | cacgatctgataccgctcta | 386 | 46 °C | 59.5 |
|  | R | tttagcttctgccagagtca |  |  |  |
| EXON 61 | F | tcaatttttcttggtaacatcg | 397 | 46 °C | 54.5/58 |
|  | R | cccttctgtcccttatcaga |  |  |  |
| EXON 62 | F | acatagagcaccctgcaaat | 372 | 46 °C | 56.5/59 |
|  | R | tctttttggcttaaataaagga |  |  |  |
| EXON 63 | F | ccaggcaggatctgaaactt | 374 | 50 °C | 54/56.5 |
|  | R | tacagccgggcttcaaaata |  |  |  |
| EXON 64 | F | atgcccagttacatccattt | 448 | 46 °C | 56/60 |
|  | R | gcacacattcttagacacacg |  |  |  |
| EXON 65 | F | cagagcaagaacctgtctca | 391 | 46 °C | 54.5/59.5 |
